# Microarray profiling to analyze the effect of Snai1 loss in mouse intestinal epithelium

**DOI:** 10.1016/j.gdata.2015.05.032

**Published:** 2015-06-03

**Authors:** Gary R. Hime, Katja Horvay, Thierry Jardé, Franca Casagranda, Victoria M. Perreau, Helen E. Abud

**Affiliations:** a Department of Anatomy and Neuroscience, University of Melbourne, Parkville, Vic 3010, Australia; b The Florey Institute of Neuroscience and Mental Health, Parkville, Vic 3010, Australia; c Department of Anatomy and Developmental Biology, Monash University, Clayton, Vic 3800, Australia

**Keywords:** Snai1, Intestine, Stem cells, SerinC3

## Abstract

Epithelial stem cells from a variety of tissues have been shown to express genes linked to mesenchymal cell states. The Snail family of transcriptional factors has long been regarded as a marker of mesenchymal cells, however recent studies have indicated an involvement in regulation of epithelial stem cell populations. Snai1 is expressed in the stem cell population found at the base of the mouse small intestinal crypt that is responsible for generating all differentiated cell types of the intestinal epithelium. We utilized an inducible Cre recombinase approach in the intestinal epithelium combined with a conditional floxed *Snai1* allele to induce knockout of gene function in the stem cell population. Loss of *Snai1* resulted in loss of crypt base columnar cells and a failure to induce a proliferative response following radiation damage. We induced *Snai1* loss in cultured organoids that had been derived from epithelial cells and compared gene expression to organoids with functional *Snai1*. Here we describe in detail the methods for generation of knockout organoids and analysis of microarray data that has been deposited in Gene Expression Omnibus (GEO):GSE65005.

**Table:** 

Specifications.	
Organism/cell line/tissue	*Mus musculus*/cultured organoids derived from small intestine
Sex	Female
Sequencer or array type	Illumina MouseWG-6_V2
Data format	Normalized data:.idat files
Experimental factors	Control vs. Snai1 knockout small intestinal organoids
Experimental features	Organoids from *VillinCreERT2 Snai1^+/+^* (control) and *VillinCreERT2* *Snai1^fl/fl^* small intestinal organoids were treated with tamoxifen to induce Cre activity and were cultured for 24 h prior to RNA extraction.
Consent	All protocols were approved by the Monash University Animal Research Platform Animal Ethics Committee
Sample source location	Clayton, Australia

## 1 Direct link to deposited data

The deposited data can be found at: http://www.ncbi.nlm.nih.gov/geo/query/acc.cgi?acc=GSE65005.

## 2 Experimental design, materials and methods

### 2.1 Mice

Snai1 is strongly localized to nuclei of crypt base columnar stem cells [1] and was likely to regulate stem cell function so experiments were undertaken to knockout Snai1 function in the mouse intestinal epithelium. Germline loss of mouse *Snai1* results in early embryonic lethality [2] thus in order to analyze the function of Snai1 in adult intestinal epithelium, a floxed *Snai1* allele, *Snai*^*fl*/*fl*^ [3], was combined with an inducible Cre allele, *VillinCreERT2* [4], that expressed CreERT2 in all cells of the small intestinal epithelium. Cre activity was induced via four daily intraperitoneal injections of 100 mg/kg tamoxifen dissolved in corn oil and on day 5 mice were killed and tissue harvested. At least 3 biological replicates of experimental, *VillinCreERT2 Snai1*^*fl*/*fl*^, and control, *VillinCreERT2 Snai1*^*+*/*+*^ mice were used to study the role of Snai1 in experiments as reported in Horvay et al. (2015) [5].

### 2.2 Generation and culture of organoids

Organoid cultures can be established from isolated crypts or individual intestinal stem cells and provide an opportunity to assay gene function in relation to stem cell function or intestinal regeneration in a defined culture system [6], [7]. This system allowed us to induce loss of Snai1 with precise temporal control, assay organoid growth and apoptosis, and collect RNA from a pure population of epithelial cells [5]. The small intestinal tract from female mice was isolated by dissection just posterior to the stomach and just anterior to the caecum following removal of associated fat and mesentery. The intestinal tract was placed in cold phosphate buffered saline (PBS), cut longitudinally and gently flushed to remove luminal contents. Villi were removed by scraping the luminal surface with a glass coverslip. The intestinal tract was then cut into small pieces in cold PBS and washed 3 times before incubation in 30 ml 2 mM EDTA in PBS with gentle rotation at 4 °C for 30 min. The supernatant was discarded and replaced with 15 ml cold PBS and mechanically dissociated by pipetting. The supernatant was again discarded and replaced with another 15 ml of cold PBS. This process was repeated with crypt containing fractions 2 to 4 collected. Crypts were pelleted by centrifugation at 1500 rpm for 3 min, the supernatant discarded and the pellet resuspended in cold DMEM/F12 (Invitrogen 21,331,046) and passed through a 70 μm cell strainer (BD 356231). The resulting supernatant was gently centrifuged at 700 rpm for 2 min to remove lymphocytes. The resulting crypt preparation was checked and counted under the microscope, the supernatant discarded and the pellet containing crypts resuspended in phenol red free Matrigel (BD 356231). 50 μl of Matrigel was seeded in each well of a pre-warmed 24 well plate and incubated for 5 min until well solidified. 500 μl of crypt culture medium (DMEM/F12 supplemented with B27, glutamax, N2, 10 mM HEPES, fungizone, 50 ng/ml EGF (Peprotech), 100 ng/ml Noggin, (Peprotech), penicillin/streptomycin and 600 ng/ml R-spondin 1 (R&D systems)) was added to each well. After several days in culture, seeded organoids would start to form buds or crypt like domains. Medium on organoid cultures was changed 3 times per week. Following 7 to 10 days in culture, organoids were passaged by mechanically disrupting the organoids in Matrigel by pipetting. 150 crypt fragments were reseeded into 50 μl of Matrigel and placed in culture medium. *Snai1* deletion was induced by adding 0.5 μM Tamoxifen (Sigma) in the culture medium to cultures established from *VillinCreERT2 Snai1*^*fl*/*fl*^, compared to control, *VillinCreERT2 Snai1*^*+*/*+*^ mice. For microarray studies, organoids were harvested 24 h following exposure to the drug.

### 2.3 Extraction of RNA and generation of cDNA

RNA from mice was prepared from isolated crypts by incubation in PBS-EDTA using similar methodology to that described for preparation of organoid cultures. 1 ml of TRIzol Reagent (Invitrogen) was added to 100 mg of isolated crypts and mechanically lysed by pipetting up and down several times. Following addition of 0.2 ml of chloroform and vigorous shaking the tubes were centrifuged (12,000 ×* g* at 4 °C) for 15 min. The upper aqueous phase was removed and RNA precipitated by adding 0.5 ml of isopropanol. The RNA pellet was washed in 70% ethanol before being dissolved in 50 μl RNase free water. Further RNA cleanup and DNase digestion was achieved using the RNeasy mini kit from Qiagen.

After 24 h of tamoxifen treatment, the matrigel-embedded organoids were gently mechanically disrupted by pipetting, washed with PBS and centrifugated at 1500 rpm for 3 min. The supernatant was discarded and the pellet containing organoids was resuspended in RLT lysis buffer (Qiagen RNeasy Micro kit). The solution was then homogenized using QIAshredder columns (Qiagen) and extraction of RNA was subsequently achieved following manufacturer's recommendations (Qiagen RNeasy Micro kit).

cDNA was prepared from epithelial crypt preps (1 μg RNA) or organoid cultures (0.5 μg) using Superscript III Reverse Transcriptase (Invitrogen) with random hexamers or QuantiTect Reverse Transcription Kit (Qiagen).

### 2.4 Microarray analysis

Labelling and hybridization of RNA was conducted by the Australian Genome Research Facility. Total RNA quality and quantity was assayed with an Agilent Bioanalyser 2100 using the NanoChip protocol. 500 ng of total RNA from each sample was used to prepare a probe cocktail (cRNA @ 0.05 μg/μl). 30 μl of total hybridization volume for each sample was loaded into a single array on the *Ilumina MouseWG-6*_*V2 Expression Beadchip*. The chip was hybridized at 58 °C for 16 h. Chips were then washed as per the Illumina manual and coupled with Cy3 and scanned in an Illumina iScan Reader. The lumiR package of R Bioconductor was applied to raw signal intensity values to conduct background correction, log2 transformation and variance stabilization. Partek Genomics Suite™ software was used to perform ANOVA of normalized probe intensities and calculate significance of variation between groups. The SerinC3 gene was found to be downregulated at 24 h after induction of *Snai1* loss [5]. Little is known of SerinC3 (also known as TDE1) function except that is has been associated with protection from apoptosis [8]. Loss of *Snai1* resulted in apoptosis of the crypt base columnar stem cell population. Down regulation of SerinC3 was confirmed by quantitative PCR of RNA isolated from organoids 24 and 72 h post treatment with tamoxifen [5].

### 2.5 Droplet digital PCR

**Fig. 1:**
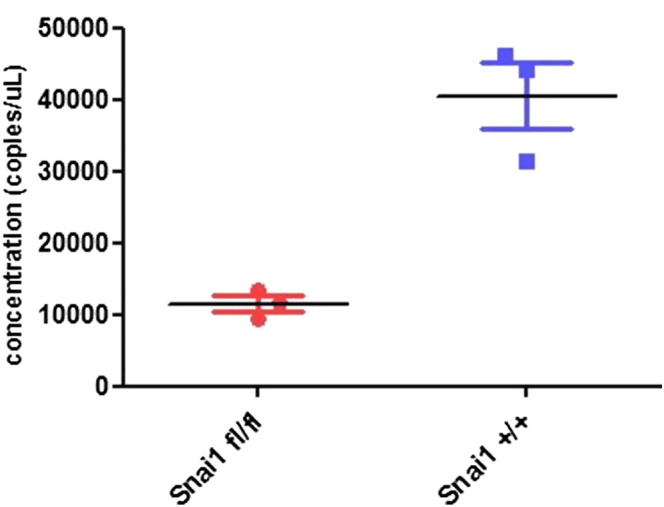
Droplet digital PCR of SerinC3 (normalized to HPRT) expression in *VillinCreERT2 Snai1*^*fl*/*fl*^ intestinal crypts, 5 days after tamoxifen treatment, compared to control crypts, *VillinCreERT2 Snai1*^*+*/*+*^*. p* = 0.004, Student's *T-*test*.*

ddPCR is a form of quantitative PCR that is based on partition of the PCR reaction mix into many thousands of droplets prior to initiation of the PCR reaction. Some droplets will contain a target molecule and some will not, hence PCR products will only be generated in a percentage of the droplets and counting these “positive” droplets allows a calculation of the number of target cDNA molecules that were present in the reaction mix. We used this sensitive method [9] to measure the expression of SerinC3 in total RNA isolated from *Snai1* mutant and control mouse small intestinal crypt preps, 5 days post treatment with tamoxifen. cDNA was generated as above. Reaction mix [2xddPCR Supermix (BioRad # 186–3010)], 20 × stock concentration of primers and probe mix (IDT PrimeTime qPCR assay 500 nM primers and 250 nM probe) and cDNA (variable volume) in a 25 μl total volume. Droplets were generated and subjected to a 2-step thermocycling protocol [95 °C × 10 min; 40 cycles x [(94 °C × 30 s, 60 °C × 60 s); 98 °C × 10 min, ramp rate set at 2.5 °C/s]. Droplet fluorescence was counted in a QX100 Droplet Reader (BioRad) and analyzed with QuantaSoft software (BioRad). The SerinC3 assay Mm.Pt.58.28709379 (IDT) was normalized with a HPRT (hypoxathine phosphoribosyltransferase) housekeeper assay Mm.PT.39a.22214828 (IDT). Expression of SerinC3 was reduced approximately 4-fold in the *Snai1* knockout crypts (Fig. 1) in a similar manner to the reduction of expression exhibited by the *Snai1* knockout organoids. The reduction in SerinC3 is thus a direct consequence of the loss of *Snai1 in vivo* within the small intestinal epithelium.
